# Endoscopic ultrasound-guided radiofrequency ablation followed by ethanol ablation of a solid pseudopapillary neoplasm of the pancreas

**DOI:** 10.1055/a-2761-0567

**Published:** 2026-01-13

**Authors:** Marcin Polkowski, Mateusz Szmit, Krzysztof Skoczylas, Jakub Krzyżkowiak, Andrzej Mróz, Jakub Pałucki, Jarosław Reguła

**Affiliations:** 1 Department of Gastroenterology, Hepatology and Clinical Oncology, Center of Postgraduate Medical Education, Warsaw, Poland; 2 Department of Oncological Gastroenterology, The Maria Skłodowska-Curie National Research Institute of Oncology, Warsaw, Poland; 3 Department of Pathology, The Maria Skłodowska-Curie National Research Institute of Oncology, Warsaw, Poland; 4 Department of Radiology, The Maria Skłodowska-Curie National Research Institute of Oncology, Warsaw, Poland

A solid pseudopapillary neoplasm (SPPN) is a rare pancreatic tumor that predominantly affects young females and typically presents as a large mass with a cystic component. With the increasing use of diagnostic imaging, small SPPNs (≤2 cm) are now detected more frequently. Although their natural history remains unclear and malignant potential is considered low, surgical resection is often recommended 1 . Recently, several cases of small SPPNs successfully treated with endoscopic ultrasound (EUS)–guided radiofrequency ablation (RFA) have been reported 2 3 . We describe an additional case managed with EUS-RFA followed by ethanol ablation (EA) for a residual tumor post-RFA.

**Fig. 1:**
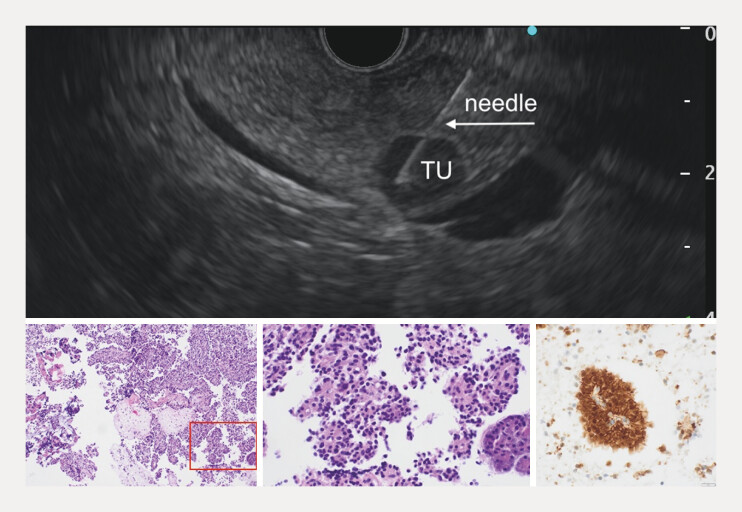
Fine-needle biopsy of the tumor (TU) with a 25G needle (upper panel). Histopathological images (lower panel, from left to right) show the hematoxylin and eosin (H&E) low-power view demonstrating pseudopapillary structures composed of uniform tumor cells; H&E high-power view; immunostaining for β-catenin demonstrating strong nuclear and membranous expression in tumor cells.

A 38-year-old woman had a 13-mm pancreatic head nodule incidentally detected on transabdominal ultrasound and subsequently confirmed by magnetic resonance imaging. EUS-guided biopsy diagnosed SPPN ( Fig. 1 ). The patient declined surgical resection and opted for EUS-guided RFA.

**Fig. 2:**
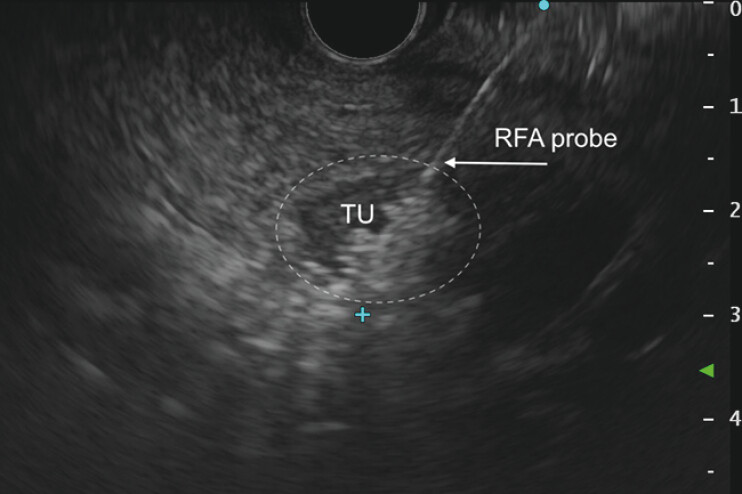
An EUS image of the tumor (TU, outlined with a dashed line) during radiofrequency ablation (RFA). The hyperechoic blush around the RFA probe in the lower right portion of the tumor represents gas microbubbles formed by tissue heating, indicating effective ablation. EUS, endoscopic ultrasound.

After EUS evaluation, a RFA probe (EUSRA, 19G, 10-mm active tip; TaeWoong Medical) was advanced under EUS guidance into the tumor. The radiofrequency energy (50 W) was delivered to three regions of the tumor in applications lasting for 8, 7, and 7 seconds, guided by EUS imaging and the rise in impedance measured using a RFA generator ( Fig. 2 , Video 1 ).

Successful EUS-guided ablation of a small (13 × 11mm) solid pseudopapillary neoplasm of the pancreatic head using radiofrequency ablation (RFA) followed by ethanol ablation of a residual post-RFA lesion. EUS, endoscopic ultrasound.Video 1

**Fig. 3:**
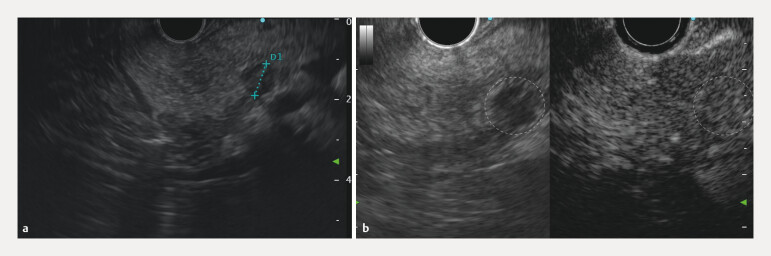
**a** An EUS image showing a residual hypoechoic lesion (between crosses), 8 × 7 mm in size, at the site of the 13 × 11 mm tumor treated with radiofrequency ablation 6 months earlier. **b** Contrast enhancement of the residual lesion indicates the viable tumor tissue. The locations of the residual lesion in both the B-mode image and the CHE image are outlined with a dashed line. EUS, endoscopic ultrasound.

**Fig. 4:**
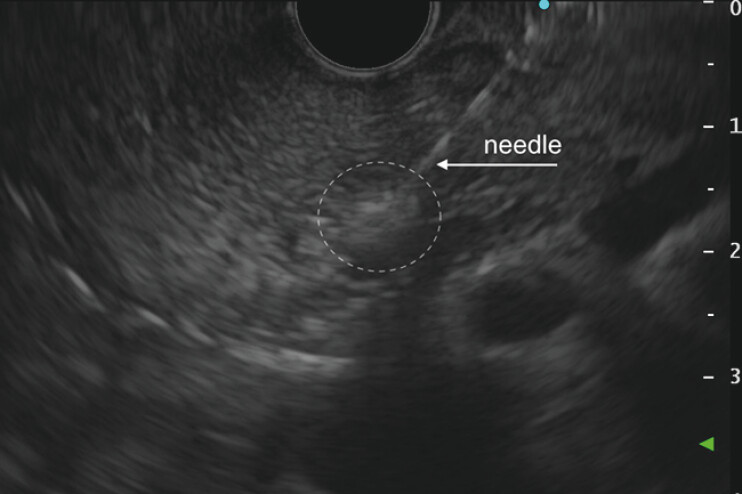
A residual post-RFA lesion (outlined with a dashed line) during ethanol injection with a 25G needle. A hyperechoic cloud around the needle tip represents ethanol spread in the tissue. RFA, radiofrequency ablation.

Follow-up EUS performed 6 months after EUS-RFA revealed an 8 × 7mm residual lesion at the treatment site ( Fig. 3 **a** ). Contrast-enhanced EUS demonstrated enhancement, suggesting viable tumor tissue ( Fig. 3 **b** ). Given the small volume of the residual lesion, EA was performed to achieve complete ablation. A total of 0.4 mL of 96% ethanol (Ethanol Sterop) was injected through a 25G EUS needle (EZ Shot 3 Plus, Olympus) in three fractions ( Fig. 4 , Video 1 ). For both RFA and EA, rectal diclofenac (100 mg) and intravenous antibiotics were administered; the post procedure course was uneventful.

Follow-up EUS and computed tomography 6 months post-EA (12 months post-RFA) showed no residual or recurrent lesion. This case adds to previous reports of successful EUS-guided ablation of SPPNs 3 .

Endoscopy_UCTN_Code_TTT_1AS_2AI
